# Leveraging large language models to construct feedback from medical multiple-choice Questions

**DOI:** 10.1038/s41598-024-79245-x

**Published:** 2024-11-13

**Authors:** Mihaela Tomova, Iván Roselló Atanet, Victoria Sehy, Miriam Sieg, Maren März, Patrick Mäder

**Affiliations:** 1 Data-Intensive Systems and Visualization Group (dAI.SY), Fakultät für Informatik und Automatisierung, Technische Universität Ilmenau, Ehrenbergstraße 29, 98693 Ilmenau, Thuringia Germany; 2 AG Progress Test Medizin, Charité - Universitätsmedizin Berlin, Corporate Member of Freie Universität Berlin, Humboldt Universität zu Berlin, Charitéplatz 1, Berlin, 10117 Berlin Germany; 3 Fakultät für Biowissenschaften, Friedrich Schiller Universität Jena, Schloßgasse 10, 07743 Jena, Thuringia Germany; 4 Institute of Biometry and Clinical Epidemiology, Charité - Universitätsmedizin Berlin, Corporate Member of Freie Universität Berlin and Humbold Universität zu Berlin, Charitéplatz 1, 10117 Berlin, Germany

**Keywords:** Large language models, Data analysis, Natural language processing, Machine learning, Feedback, Health care, Medical research, Computer science

## Abstract

Exams like the formative Progress Test Medizin can enhance their effectiveness by offering feedback beyond numerical scores. Content-based feedback, which encompasses relevant information from exam questions, can be valuable for students by offering them insight into their performance on the current exam, as well as serving as study aids and tools for revision. Our goal was to utilize Large Language Models (LLMs) in preparing content-based feedback for the Progress Test Medizin and evaluate their effectiveness in this task. We utilize two popular LLMs and conduct a comparative assessment by performing textual similarity on the generated outputs. Furthermore, we study via a survey how medical practitioners and medical educators assess the capabilities of LLMs and perceive the usage of LLMs for the task of generating content-based feedback for PTM exams. Our findings show that both examined LLMs performed similarly. Both have their own advantages and disadvantages. Our survey results indicate that one LLM produces slightly better outputs; however, this comes at a cost since it is a paid service, while the other is free to use. Overall, medical practitioners and educators who participated in the survey find the generated feedback relevant and useful, and they are open to using LLMs for such tasks in the future. We conclude that while the content-based feedback generated by the LLM may not be perfect, it nevertheless can be considered a valuable addition to the numerical feedback currently provided.

## Introduction

**Fig. 1:**
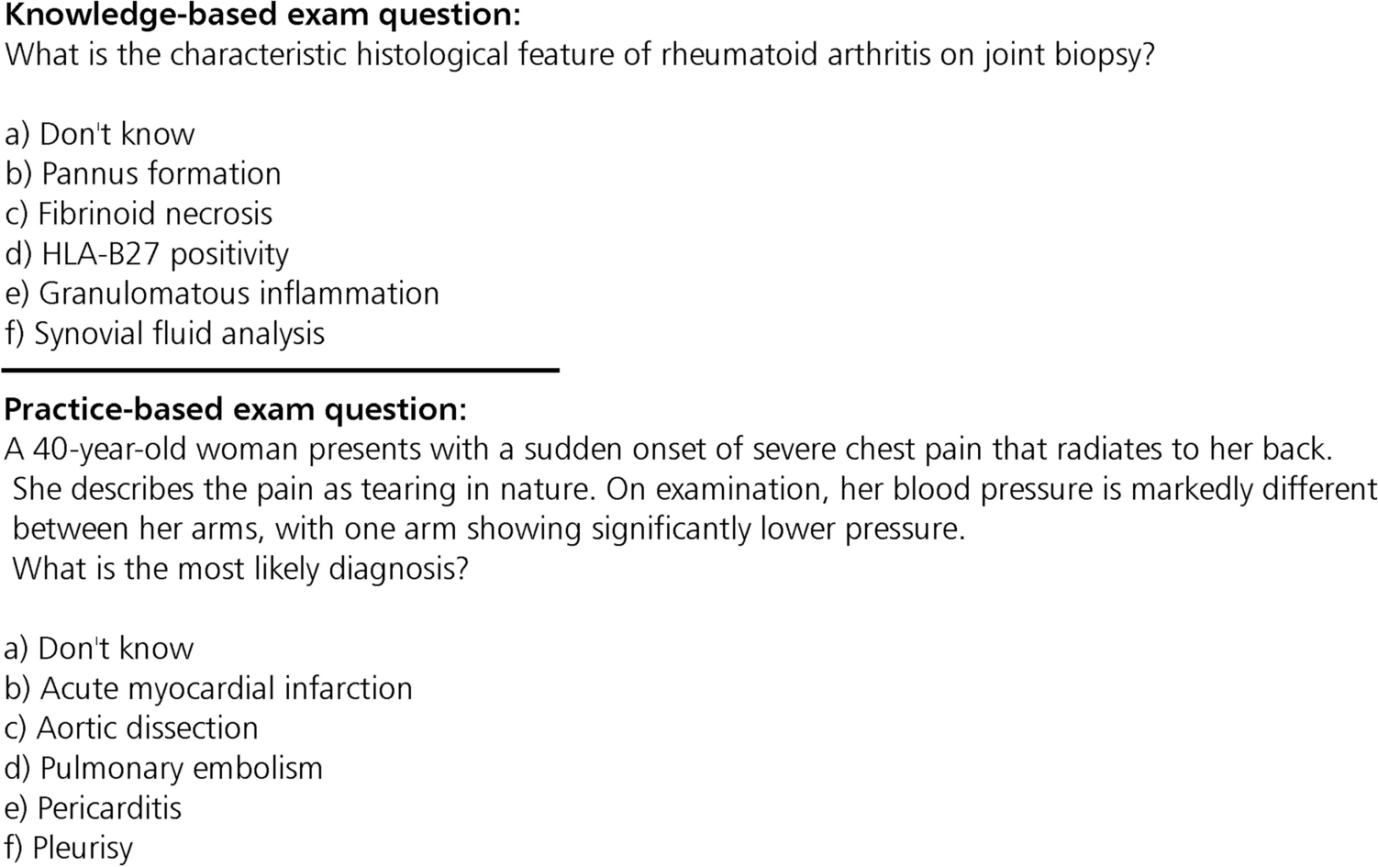
Examples of knowledge-based and practice-based PTM questions. The example questions are generated by AI due to copyright reasons of the original PTM questions.

In Germany, the ‘Progress Test Medizin’ (PTM) was jointly introduced by Charité - Universitätsmedizin Berlin (Charité) and Witten/Herdecke University in 1999 and encompasses the idea of monitoring medical students’ progress through to graduation, aiming to provide a cross-sectional and longitudinal assessment, demonstrating the growth and effectiveness of student knowledge (Ref.^1^ and references therein). Today, the PTM is administered to approximately 11,000 students at medical universities (in Germany, Austria, and Switzerland) each term. The PTM is a formative test to be completed within 180 minutes and is administered each semester. One PTM run consists of 200 questions and covers content from the entire medical curriculum, ranging from knowledge-based questions about definitions across various medical fields to more complex questions, such as correctly identifying illnesses, selecting and administering appropriate therapies, emergency measures, and medications based on given scenarios. The PTM questions for each run are carefully selected from a continuously maintained database containing more than 2000 questions. Each PTM question in the database is linked to at least one of nine domains, one of fourteen organ systems, and one of twenty-seven subjects. Domains encompass fundamental areas such as structure and function, pathogenesis, and pathomechanisms, as well as clinical aspects like diagnostics, diagnosis, therapy, emergency care, prevention, and management. Scientific, ethical, and legal dimensions are also represented by domains. Organ systems and subjects, on the other hand, are associated with the type of medical curriculum being followed, i.e., systems-based or subject-based. Systems-based medical curriculum are organized by organ system, e.g., cardiovascular system, nervous system, or life stage, e.g., paediatrics, and geriatrics. Following this curriculum, students learn all aspects of a particular system or life stage simultaneously. Subject-based medical curriculum are usually structured according to medical specialties. Following this curriculum, students can gain a deep understanding of each specialty^2^. Questions in the PTMs can also be differentiated by whether they are knowledge-based, targeting the understanding or recall of specific information on a given topic, or practice-based, targeting medical students’ ability to apply knowledge or skills in practical or real-world situations. Examples of knowledge-based and practice-based PTM questions are presented in Fig. 1.

Currently, the PTM feedback is in the form of numerical scores, providing, on one hand, a general idea of how well different domains, organ systems and medical subjects are mastered by medical students and on the other hand providing an overview of one’s performance against the performance of other peers^1,3,4^. For more details about the aim, structure, and evaluation of the PTM, we refer the reader to the work of Osterburg et al.^5^.

Feedback is an essential part of the learning process, aiming to provide students with an overview on their performance, helping them to identify problematic areas, and guiding them towards better understanding. The form and the delivery of feedback to students is an ongoing field of research^6–8^ that investigates ways to provide students with better feedback and assesses the quality and effects of different types of feedback on students^3,9^. Studies have shown that students desire additional feedback beyond whether their answers are correct and incorrect, and would like it to be more detailed and specific, showcasing for example their strengths and weaknesses^3,10^. Furthermore, the way feedback is provided is shown to have an effect on students’ motivation, learning, and achievements^11,12^. Thus, it is important to construct and present feedback in a manner that promotes learning and retention of information, as well as to act as a guideline to students in their further studies. We thus see the need, in addition to numerical scores, to provide medical students with further feedback, tailored from the content of test questions. Such content can encompass key topics in a PTM question and important information associated with these topics. This kind of feedback we term in the manuscript as content-based feedback. While the current feedback in the PTM provides an overview of how well separate domains, organ systems, and subjects are understood, it offers only a broad perspective on knowledge gaps. This is because each domain, organ system, and subject encompasses numerous specific medical topics and concepts. This can lead to students, especially those who are in their early years of study, being uncertain about which specific areas require further study and improvement. Content-based feedback, which highlights important information from the questions, can be especially useful in this scenario. Additionally, in cases where questions cannot be displayed after the test-such as when they are reused periodically or to prevent memorization, as with PTM questions-a condensed form of content in the test questions can still offer an overview of the questions. When formulated appropriately, content-based feedback can offer students an overview of what specific topics were covered in a PTM. Furthermore, when combined with information about whether a question was answered correctly or incorrectly, it can highlight areas where a student is proficient in and where one needs to improve. To ensure that content-based feedback is both useful and effective, it must adhere to good feedback practices. Such feedback, as identified by David Nicol and Debra Macfarlane-Dick^13^, can help students to constitute good performance, specify goals, criteria, and expected standards, promote self-assessment during learning, and help students to bridge the gap between current and desired performance. However, constructing feedback that reflects learning goals, criteria and expected standards can be challenging for educators. Producing high-quality feedback requires time, which educators, as mentioned in the work of Blair et al.^14^, do not always have. Furthermore, extracting relevant information from PTM questions is challenging due to the limited semantic information in some questions. For example, practice-based questions can be longer, containing multiple sentences, while knowledge-based questions are often limited to a single sentence. The quality and consistency of feedback derived from test question content can be subjective and influenced by factors such as time constraints, the educator’s concentration, and the individual interpretation of what is important from the question.

Advancements in artificial intelligence (AI), i.e., in natural language processing (NLP) in the form of large language models (LLMs) present a promising solution to this intricate task, considering the amount of data such models are trained on, their capabilities of summarizing, translating, writing poetry and stories, answering questions, and providing explanations. The potential use of AI in the medical field is not a new concept, and research into how AI can be applied and for which tasks it is most effective is ongoing. For example, in the work of Xu et al.^15^, the authors developed a digital ophthalmologist app using GPT-4V and explored the extent to which a GPT-4V(ision)-based chatbot can interpret multimodal input, consisting of an image and related questions to the image, in interpreting ocular multimodal images. Their work serves as a benchmark for enhancing ophthalmic multimodal models. Similarly, the work of Choi et al.^16^ uses ophthalmological data (oculomics) in combination with regression models developed by ChatGPT-4 to predict the risk of osteoporosis, aiming to enable more effective prevention strategies and to provide treatment tailored to individual patients. How and for what to utilize AI in education is an also actively researched topic. In the work of Cheung et al.^17^ the applicability of the LLM ChatGPT to generate multiple-choice questions (MCQs) in graduate medical examinations is assessed and compared against MCQs formulated by experienced university professors. The assessment showed no significant difference in question quality produced by both the LLM and the university professors, suggesting their utilization to assist educators when drafting exam questions. The work of Kazemitabaar et al.^18^ studied the possible use of the AI code generator OpenAI Codex in the educational domain to assist students in learning programming. The results from their experiment shows that students who used Codex to solve a set of 45 programming tasks were less frustrated and more motivated than students without access to Codex. Furthermore, the Codex-group showed better retention on post-tests conducted 1 week later, highlighting the efficacy of AI in helping students to learn to code. The previously discussed studies use LLMs to assist educators in generating MCQs for a medical undergraduate exam. However, there is almost no previous work on their utilization in generating feedback from medical multiple-choice exam questions. Feedback generated by artificial intelligence is considered a new branch of research and is still not well represented in literature. The work of Meyer et al.^19^ utilized LLMs to generate feedback on written assignments for the Test of English as a foreign language (TOEFL iBT®). In our work, compared to the work of Meyer et al., we aim to generate content-based feedback, specifically from medical multiple-choice exam questions, by utilizing two prominent LLMs, i.e., ChatGPT 4.0^5^ by OpenAI and Bing Chat^2^ by Microsoft. Furthermore, the feedback provided in the work of Meyer et al. focuses on improving writing by providing hints and guidelines about the structure, content, and language used by a student. In our case, we aim to extract essential information from a short piece of text that covers a variety of medical topics, directly or indirectly mentioned in the text, while providing additional information associated with the identified medical topics.

Before leveraging LLMs for generating feedback from PTM questions, it is necessary to first identify what the feedback should include and how it can be effectively utilized by students. The aim of feedback, in general, is to provide information about knowledge proficiency and deficiencies, while promoting learning and retention of information^13^. The works of Blair et al.^14^, Sehy et al.^20^, and Gray et al.^21^ indicate that students desire more specific feedback rather than the typically provided right-and-wrong feedback. Therefore, feedback should deliver high quality information about students’ learning^13^ and should summarize important information, necessary for a student to know and understand in order to answer the posed question. Furthermore, students find written feedback useful as they can refer to it later and learn from it^14^. Therefore, feedback should provide opportunities to improve one’s knowledge and should promote the ability to evaluate one’s own learning through reflection^13^. However, research shows that not all written feedback is perceived well by students. Gal et al.^22^ observed in their work that this is not the case with longer written feedback. Thus, feedback should exclude overly specific information and be moderate in length to avoid overwhelming students. Additionally, feedback is expected to be clear, and easy to understand^14^.

**Fig. 2:**
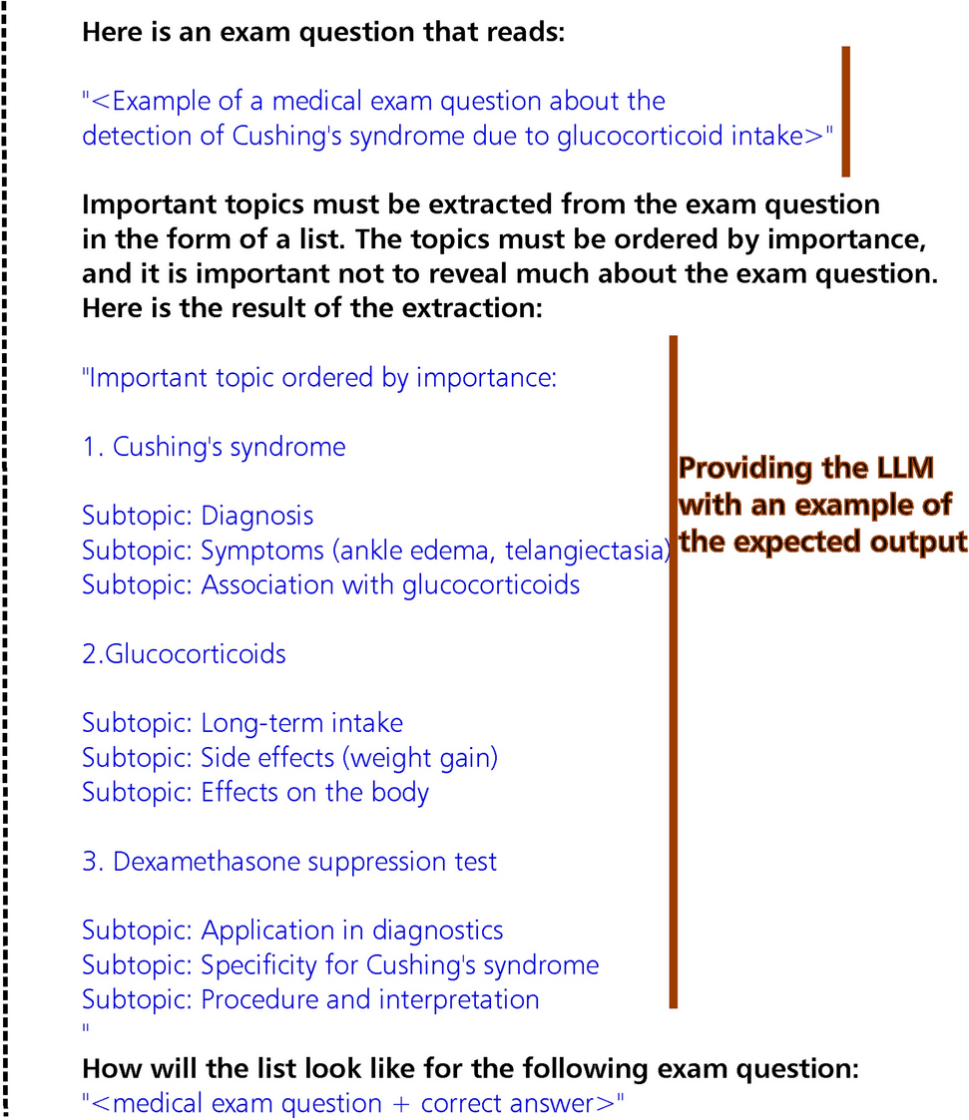
Prompt used to generate content-based feedback. Original prompt was in German.

When working with LLMs it is important to consider how to prompt them. Prompts can be considered as guiding points for a model during response generation. They serve as instructions, outlining the content of the response, the degree of the details, the style of writing and the format of the response. The prompt we formulated followed feedback principles found in literature. Specifically, the feedback principles we followed are that feedback should be specific and summarize important information^13,14,20^, feedback should be written for use in later revision^14^, and feedback should be clear and easy to understand^14,22^. In Fig. 2, we present the prompt we used.

Our prompt provides useful information in the form of topics and subtopics without exposing too much detail from the question. Subtopics provide additional information about the topics. Furthermore, considering the performance of students on an exam, the topics could give them a general overview of which topics they were proficient in and which topics they were deficient in. The subtopics could be used as a way to review information about the topics. In cases where students were not familiar with a topic, the subtopics could serve as guiding points for learning what is important for this specific topic. Additionally, presenting the response in a structured form allowed it to be presented more clearly.

Giving the effects of feedback on students and the importance of providing qualitatively good feedback, we aim to address in our manuscript the following research questions:

1. *How do the results generated by LLMs, i.e., ChatGPT 4.0 and Bing Chat compare to each other?* We address this RQ by comparing the length and the textual similarity between the generated outputs by the LLMs ChatGPT 4.0 and Bing Chat. Furthermore, we conduct a survey with medical experts and educators familiar with the PTM.
2. *Are the LLMs utilized for extracting important information from PTM questions useful?* We address this RQ using insights gained from the same survey conducted in RQ 1, which targeted medical experts and educators familiar with the PTM.

In particular, our study makes the following contributions: it is the first (1) to propose a process for applying and comparing LLMs, specifically ChatGPT 4.0 and Bing Chat, for generating content-based feedback in German from PTM questions, (2) to evaluate the content-based feedback produced by both models by comparing the outputs at a semantic level and assessing the quality of the feedback with medical practitioners and educators, and (3) to contribute to the educational field, specifically in medical education, by proposing a way to assist educators in preparing feedback from exam questions more quickly, which pinpoints important information without giving away the entire questions.

## Methods

**Fig. 3:**
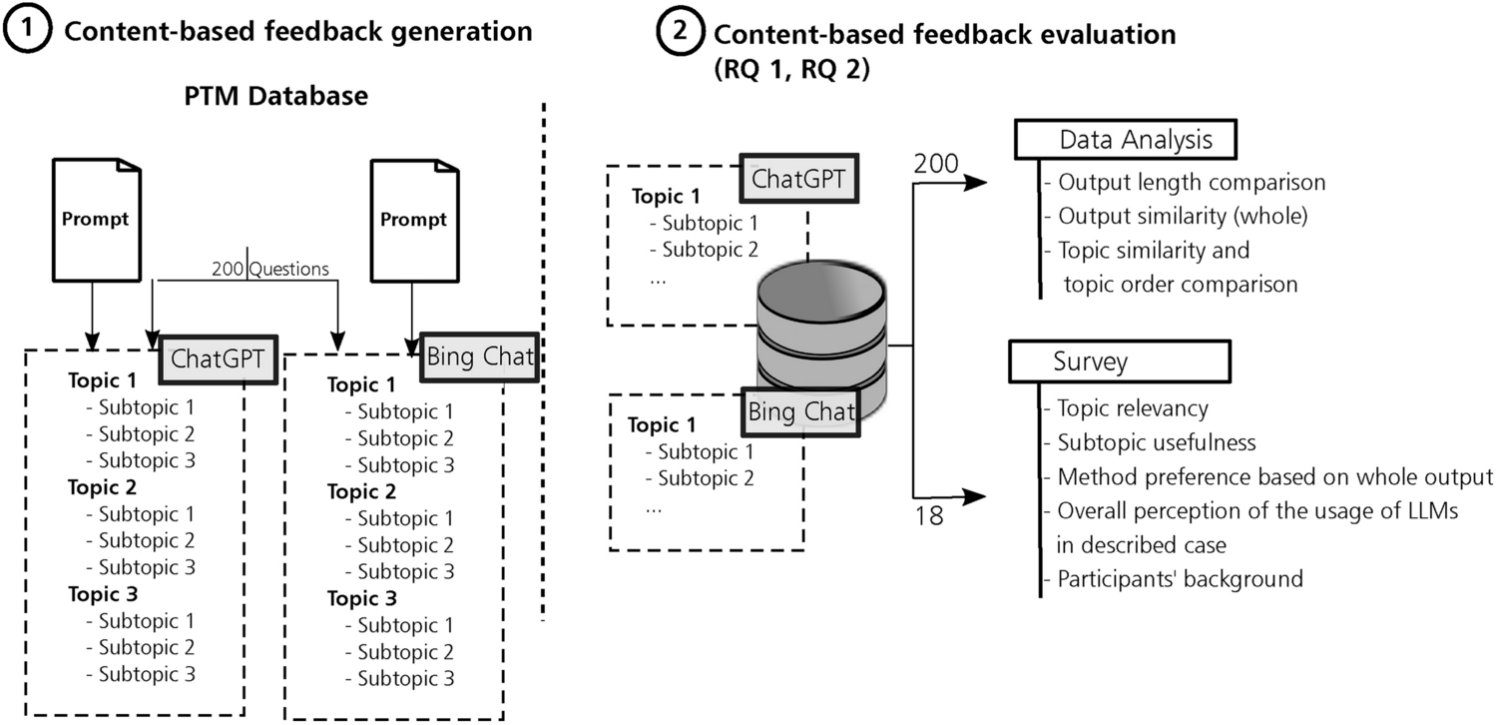
Overview of the approach utilized to generate content-based feedback and the methodology followed to evaluate the generated feedback.

To evaluate the capabilities of LLMs to generate content-based feedback from PTM questions, we followed the two-step approach proposed in Fig. 3 using two prominent LLMs, i.e., Bing Chat and ChatGPT 4.0.

ChatGPT is developed by OpenAI^23^ and was first introduced on November 30, 2022. Its architecture is based on the Generative Pre-trained Transformer (GPT). Different GPT versions exist, with the most recent, at the time of writing this manuscript, being GPT-4. OpenAI provides various ways to interact with their GPT models, ranging from using different GPT versions via an API in a chat format (known as ChatGPT) to a playground and as an API to be integrated into one’s own application. The experiments performed in this manuscript are based on GPT-4, used in a chat format. At the time of writing, ChatGPT 4.0 is a paid subscription version. Bing Chat^24^, currently known as Copilot, is an AI-powered search engine option introduced by Microsoft in Edge browsers in February 2023. The inner workings of Bing Chat are based on an OpenAI GPT LLM and the Microsoft Prometheus model, which is a proprietary model for working with the OpenAI LLM^25^. The version used by Bing Chat, at the time the experiments were performed, utilized GPT-4. At the time of writing this manuscript, Bing Chat provides three modes: creative, balanced, and precise. The creative mode, as the name suggests, is for creative writing and is the least precise mode. In contrast, the precise mode offers generated responses with the most detail and the highest precision. The balanced mode is a compromise between the two other modes^26^. The experiments performed in this manuscript were conducted using the precise mode. Both models used in this manuscript are based on GPT-4. GPT-4 is based on the transformer architecture^27^ and is pre-trained to predict the next token. The data used to train GPT-4 encompasses publicly available data, including internet data and data licensed from third-party providers^28^. Training incorporates Reinforcement Learning from Human Feedback (RLHF)^29^. RLHF uses human preferences as a reward signal during fine-tuning.

The first step in our approach involves generating content-based feedback with the prompt we presented in Fig. 2. The prompt creation followed the following process: First, we identified principles from literature that describe the expectations of students regarding the content and structure of feedback. The feedback principles we identified were: the output should be specific and summarize important information^13,14,20^, feedback should be written in a way that allows for revision at a later time^14^, and feedback should be clear and easy to understand^14,22^. Afterward, guided by these principles, we created an example of how we envision the output to look. By formulating topics and subtopics as content-based feedback instead of complete sentences, we ensured a more structured summary of the important information mentioned in the questions. More structured feedback enhances clarity and makes it easier to understand. Lastly, we provided the models with an example of how we expect the output to look like as to ensure that they can follow instructions and produce consistent responses based on the provided prompt. The second step involves comparing and assessing the outputs generated by both LLMs. To compare the examined LLMs, we performed data analysis on the outputs generated by both models. To evaluate the correctness and usefulness of the content-based feedback generated by the LLMs, we administered a survey containing a set of the generated feedback to medical educators and practitioners.

### Generating content-based feedback from PTM exam questions

With the prompt we formulated for this task, we generated content-based feedback with ChatGPT 4.0^23^ and Bing Chat^24^ for 200 PTM exam questions. At the time of performing our experiment, the version of Bing Chat we employed utilized GPT-4.0. Additionally, we used the precise mode to generate content-based feedback from the 200 PTM questions, as it offers the most detail and most precise responses in contrast to the other modes (the creative mode and the balance mode)^26^.

### Assessment workflow of the generated content-based feedback

The generation was performed in mid-November 2023. We did not fine-tune or train the models on PTM content. However, we tested their capabilities in answering PTM questions to generate content-based feedback, similar to the work of Friederichs et al.^30^. In their study, Friederichs et al. assessed the capabilities of ChatGPT 3.5, the predecessor of ChatGPT 4.0, on 400 PTM questions, two-thirds of which were answered correctly. In our case, both models were able to answer all 200 questions correctly. The generated content-based feedback, we then assessed by first performing data analysis using Python (3.9.18) and the Python library scikit-learn (1.4.1) (sklearn), and then by administrating a survey on the usage and usefulness of LLMs in generating content-based feedback. While the data analysis was performed on the generated feedback for the entire set of 200 PTM questions, the survey covered a subset of eighteen PTM questions, two per each domain, and their respective feedback produced by the LLMs.

*Comparing the produced content-based feedback by performing data analysis.* To compare the generated feedback from both LLMs, addressing RQ 1, we first analysed the length of the produced outputs. Then, we computed the textual similarity: once on the entire produced outputs, once focusing only on the generated topics and their order in the outputs, and once focusing on the generated subtopics. Computing the length of each output allows us to assess the level of detail in each. Computing the textual similarity between the generated outputs allows us to evaluate the extent to which the outputs produced by the two LLMs resemble each other. The generated output by the LLMs provides information in the form of topics and subtopics. The difference between topics and subtopics is that topics are directly associated with the content in a PTM question, while the information in the subtopics is not necessarily part of the content in a PTM question. Focusing only on the topics, we first compute the textual similarity between them to observe how similar the generated topics are to each other. Afterwards, we examine, based on the computed similarity scores and the order of the topics, whether topics that are similar to each other are also produced in the same order. This assessment helps determine if both LLMs prioritize topics in a similar manner, given that the two LLMs were prompted to generate topics based on an exam question and to order the topics according to their importance. To compute the length of each output, we first tokenize the generated outputs by space and then count the number of tokens. To measure textual similarity, we use cosine similarity. Cosine similarity measures the similarity between two texts (documents) represented as vectors by computing the cosine of the angle between them^31^. To obtain the vectors, the target texts need to be tokenized and preprocessed, e.g. applying lemmatization. Based on the obtain vectors, we compute the cosine similarity using the following formula:

1 $$\begin{aligned} \text {cosine}\_\text{similarity}(\textbf{a}, \textbf{b}) = \frac{\textbf{a} \cdot \textbf{b}}{\Vert \textbf{a}\Vert \cdot \Vert \textbf{b}\Vert }. \end{aligned}$$

Cosine scores can range between zero and one, with scores near zero indicating that the vectors are less similar, and scores near one indicating that the vectors are more similar.

*Survey on usage and usefulness of LLMs for extracting important information from PTM questions.* Additionally, we performed a user survey evaluating how LLMs compare to each other, how useful they are for extracting important information from PTM questions, and whether they can be viewed as helpful assistance in preparing content-based feedback.

Before conducting the survey, we asked PTM makers to act as a pilot and to perform the survey in its initial form. The PTM makers were tasked with assessing the complexity of the survey questions for the participants and the interpretability of the survey question descriptions, considering the allotted time for our participants. Based on insights from the pilot phase, we modified the survey by reducing the number of questions and refining the descriptions of the survey questions. Next, we reached out to people familiar with the PTM via email. These potential participants were individuals working in healthcare or in healthcare education, encompassing both academia and healthcare practitioners. In the email, we provided a generic link to the anonymous online survey. The survey was conducted in German. The estimated time for taking the survey was no more than 30 minutes, and the survey was accessible for a period of four weeks.

Our survey was split into two sections. In the first section, survey participants needed to evaluate the generated outputs of the examined LLMs, given the question stem and correct answers of eighteen PTM questions. We aimed to present our survey participants with a variety of questions covering different medical areas, specifically ensuring representation from the nine domains included in the PTM. These nine domains cover topics related to diagnosis, emergency recognition and measures, therapy, health promotion, prevention, structure and function, ethics, history, law, medical-scientific skills, diagnostics, and pathogenesis/pathomechanisms. In total, we randomly selected 18 PTM questions, ensuring that each of the nine domains was represented by two questions. The models’ names were hidden from the participants and instead were referred to as *Method 1* and *Method 2*. The generated output from both models was evaluated based on the following questions translated into English for this manuscript: “How relevant do you find the topics by Method 1 and Method 2 to the content of the given texts?[...]”, “Rate the usefulness of the subtopics generated for each topic[...]”, “Considering both the topics extracted and the subtopics proposed, the results of which method do you prefer overall?” A German version of the survey questions is provided as Supplementary Material. With those survey questions, we aimed to understand the two examined LLMs and get a feeling how participants perceive the generated output from each method. This survey section aimed to answer RQ 2 by using human evaluators to assess the semantic accuracy of the outputs of both examined LLMs.

In the second part of our survey, we aimed to learn more about the background of the participants and their general perception of the LLMs after assessing the performance of the LLMs. The second part included the following questions translated into English: “Would you use a large language model to extract key information from exam content after seeing the results?”, “How important is the review of the results?”, “Are you a medical practitioner?”. Additionally, we provided participants with an option to give us more feedback by answering the optional question: “Do you have any recommendations or insights regarding the utilization of LLMs for delivering content-based feedback to students, derived from examination questions”.

## Results

### Assessment of the generated content-based feedback (data analysis)

**Fig. 4:**
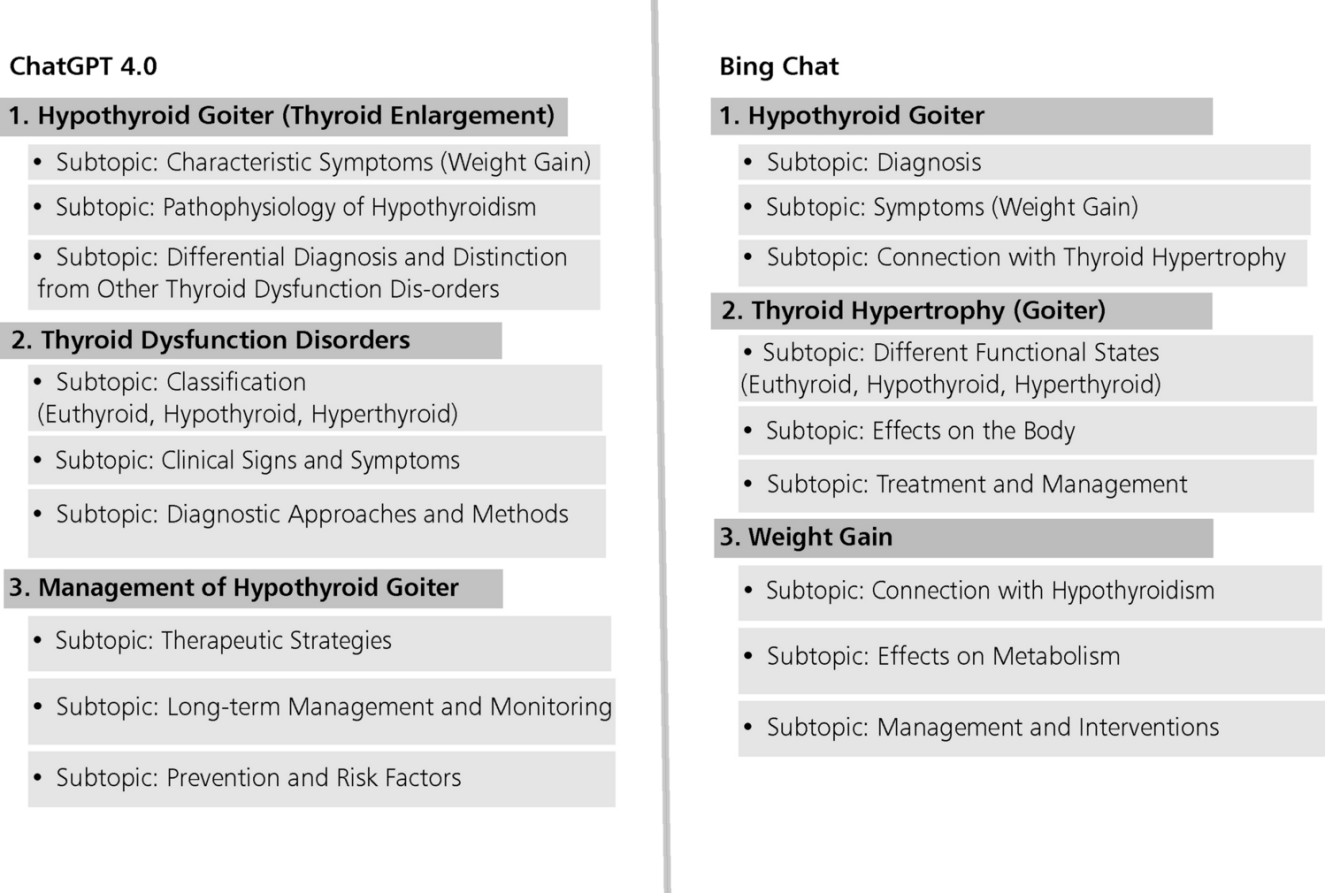
Examples of generated outputs by ChatGPT 4.0 and Bing Chat. The original outputs were in German, where the similarity score between the two is below 40%.

First, we computed the average output length produced by both examined LLMs based on word count. ChatGPT 4.0 generates slightly longer texts (41.81 words) than Bing Chat (40.23 words). Second, we computed the similarity of the content in the generated outputs by calculating the cosine similarity for each question, considering only topics, only subtopics, and both combined. We then grouped the questions by domain and calculated the average cosine similarity per domain (see Table 1). Our computation indicates that, on average, the similarity of the topics extracted by both models is around 45%, the similarity of the subtopics is around 55%, and the similarity considering both topics and subtopics is around 61%. To examine the differences among the two models, we manually compare the similarity of all generated outputs of questions, for which the pairwise similarity score was lower than 40%. In all six diverging cases, we observed that both outputs had the same medical focus and referred to roughly the same medical topics. However, outputs differed in specificity regarding subtopics. For instance, in one of the generated output pair, which is 38% similar (see Fig. 4), both models produced topics related to thyroid conditions. In the first case, the model addressed thyroid dysfunction, whereas the second focused on thyroid appearance. Additionally, the subtopics for the topic “Hypothyroid Goiter” differed in specificity, i.e., wording and content varies. The first model generated a subtopic labeled as “Characteristic Symptoms (Weight Gain)”, while the other more generically labeled it as “Symptoms (Weight Gain)”, the difference being a single additional word. Another point of divergence among the two generated outputs being that one subtopic addresses the “pathophysiology of hypothyroidism,” while the other focuses on its “diagnosis”. A further reason for the difference in similarity between the generated outputs was that, in some cases, such as Topic 3 in both generated outputs in Fig. 4, the topics differed, leading to variations in their subtopics.

**Fig. 5:**
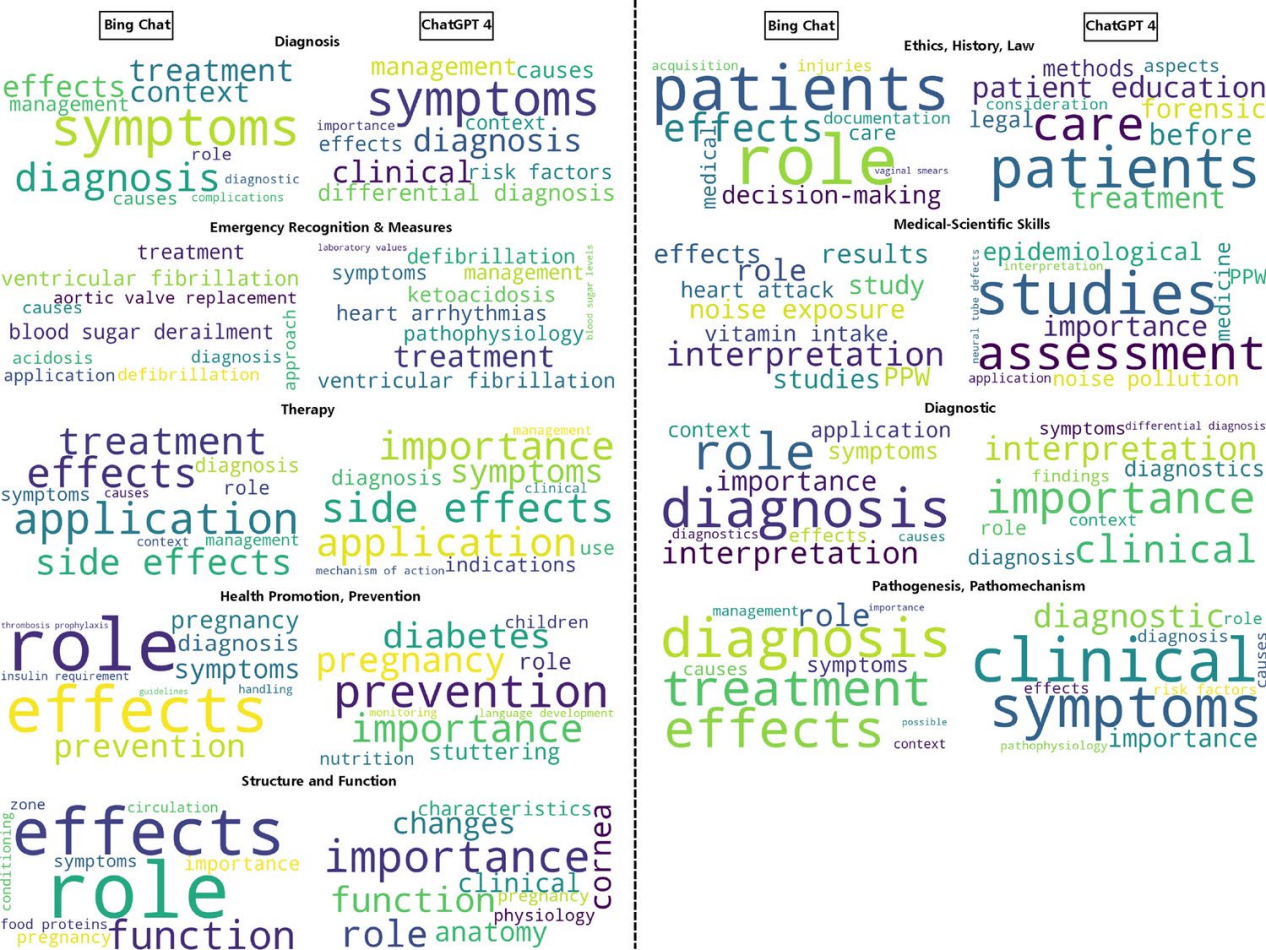
Wordclouds of the top ten words from subtopics per domain and per LLM. The words in the wordclouds are translated into English, originally they are in German.

In the case of ‘Emergency Recognition and Measures’, we observed a significantly low similarity between the topics, suggesting that the topics extracted by both models differ in content and/or wording. Overall, the similarity scores indicate that while there are similarities, there is also notable variance between the topics and subtopics. Additionally, we generated wordclouds of the top ten mentioned words (see Fig. 5), translated from German to English, for the subtopics in each domain for each LLM. By examining the words in the wordclouds produced by both models, we observe, in congruence with the computed cosine scores, some similarity in the words considered most important by both models. The complete wordclouds in German can be found in the supplementary materials.

**Table 1:** Average similarity score per domain for topics only, subtopics only, and both.

Domains	# of Qs out of 200	cos sim.-topics (AVG)	cos sim.-subtopics (AVG)	cos sim.-topics and subtopics (AVG)
Diagnosis	67	0.41	0.55	0.59
Emergency Recognition and Measures	2	0.14	0.57	0.58
Therapy	31	0.55	0.49	0.57
Health Promotion, Prevention	8	0.49	0.54	0.60
Structure and Function	13	0.52	0.6	0.66
Ethics, History, Law	3	0.47	0.52	0.59
Medical-Scientific Skills	4	0.42	0.58	0.63
Diagnostic	28	0.53	0.55	0.63
Pathogenesis, Pathomechanisms	44	0.55	0.54	0.62

Third, we focused only on the topics and the order in which they were generated. In all 200 generated outputs, ChatGPT 4.0 consistently generated three topics per PTM question. However, this was not always the case for Bing Chat; for twenty of the 200 PTM questions, Bing Chat generated only two topics. After computing the cosine similarity based only on the generated topics, we found that on average, 1.9 topics match per generated output, and on average, 1.3 topics from the matching topics are generated in the same order. This indicates a moderate level of consistency in both the content and the sequence of the topics generated. This is notable because we prompted our models to extract topics and to order them by importance. In four of the generated outputs, the topics and their order fully match, while in two of the generated outputs, the topics differ completely.

### Assessment of the generated content-based feedback (survey)

*Participants’ background.* In total, seven participants took part in our survey. Six of the participants completed the survey entirely, while one participant completed the evaluation for only half of the PTM questions. For one participant, we do not have any data indicating whether they are a medical practitioner or not. Four participants were medical practitioners, and two were healthcare educators.

*Assessing the LLMs performance across the generated topics and subtopics.* Participants rated, on average, the relevance of topics across all domains (see Table 2), using a Likert scale from 1 (low) to 5 (high). Ratings for Bing Chat ranged between 3.6 and 4.3, and for ChatGPT 4.0, ratings ranged between 3.9 and 4.9. Computing the mode for the ratings given by all participants, see Table 2, we observed that the majority rated the relevance of topics as four in the case of Bing Chat, and as 5 in the case of ChatGPT 4.0.

**Table 2:** Average ratings and mode computed for each participant to assess the relevance of topics and the usefulness of subtopics for both Bing Chat and ChatGPT 4.0. $P_{x}$ stands for participant x.

	$P_{1}$	$P_{2}$	$P_{3}$	$P_{4}$	$P_{5}$	$P_{6}$	$P_{7}$
**Bing Chat**							
Relevance topics (AVG per participant)	4.1	4.2	4.2	4.3	3.6	4.2	4.2
Relevance topics (Mode per participant)	4	4	4	5	4	5	5
Usefulness subtopics (AVG per participant)	4.3	4.7	4.1	4.1	3.2	4.2	4.0
Usefulness subtopics (Mode per participant)	4	5	4	4	3	4	4
**ChatGPT 4.0**							
Relevance topics (AVG per participant)	4.2	4.9	3.9	4.5	3.9	4.7	4.6
Relevance topics (Mode per participant)	5	5	4	5	4	5	5
Usefulness subtopics (AVG per participant)	4.4	5.0	3.8	4.2	3.6	4.4	4.4
Usefulness subtopics (Mode per participant)	4	5	4	5	4	4	4

Table 3 summarizes the average participant rating per domain regarding the relevance of the extracted topics from the PTM questions.

**Table 3:** Average ratings per domain provided by each participant regarding the relevance of topics for both Bing Chat and ChatGPT 4.0.$P_{x}$ stands for participant x.

Domains	$P_{1}$	$P_{2}$	$P_{3}$	$P_{4}$	$P_{5}$	$P_{6}$	$P_{7}$	Total (AVG)
**Bing Chat**								
AVG Diagnosis	5	4	4.5	5	3.5	3.5	3.5	4.1
AVG Emergency Recognition and Measures	5	4.5	4	4.5	4.5	4.5	5	4.6
AVG Therapy	4	4	4.5	5	3.5	4.5	4.5	4.3
AVG Health Promotion, Prevention	4	4.5	4	5	4	5	4	4.4
AVG Structure and Function	4	4.5	4	4	3.5	–	4	4
AVG Ethics, History, Law	4	5	4	5	4.5	–	4	4.4
AVG Medical-Scientific Skills	3.5	4	4	4	2	4	3.5	3.6
AVG Diagnostic	3.5	3.5	4.5	3.5	3	–	5	3.8
AVG Pathogenesis, Pathomechanisms	4	4	4.5	3	4	–	4	3.9
**ChatGPT 4.0**								
AVG Diagnosis	4.5	5	3.5	5	4.5	4.5	5	4.6
AVG Emergency Recognition and Measures	4	5	2.5	4	3	5	3	3.9
AVG Therapy	4.5	5	4.5	5	4	4	4.5	4.5
AVG Health Promotion, Prevention	5	4.5	3.5	5	4	5	4.5	4.5
AVG Structure and Function	3.5	5	4	4.5	4	–	5	4.3
AVG Ethics, History, Law	4	4.5	3.5	4.5	3.5	–	5	4.2
AVG Medical-Scientific Skills	4	5	5	4.5	4.5	5	4.5	4.6
AVG Diagnostic	4.5	5	4.5	4	3.5	–	5	4.4
AVG Pathogenesis, Pathomechanisms	3.5	5	4.5	4	4	–	5	4.3

Overall, the topics in five out of nine domains—“Diagnosis”, “Therapy”, “Health Promotion, Prevention”, “Structure and Function”, and “Ethics, History, Law”—each represented by two PTM questions, received an overall average rating of four or above from all participants using both methods. For the domain “Emergency Recognition and Measures”, Bing Chat received an average rating of four or above from all participants resulting in an overall average score of 4.6. ChatGPT 4.0, on the other hand, received an average rating below four (3.9), with only two participants giving a rating above four, and all other giving it an average rating of four or below. The difference between the overall averaged scores of the two models was 0.7. This notable difference in scores across the domain indicates significant content variation observed during our data analysis. In three domains, i.e., “Medical-Scientific Skills”, “Diagnostic”, and “Pathogenesis, Pathomechanisms”, ChatGPT 4.0 received an overall average rating of four or above from all participants, whereas Bing Chat received ratings below four. Specifically, the differences in average scores were 1.0, 0.6, and 0.4 respectively, with scores of 4.6, 4.4, and 4.3 for ChatGPT 4.0 compared to 3.6, 3.8, and 3.9 for Bing Chat.

Our participants rated, on average using a Likert scale from 1 to 5, the usefulness of subtopics in all domains (see Table 2). Ratings for Bing Chat ranged between 3.2 and 4.7, while for ChatGPT 4.0 they ranged between 3.6 and 5, with the highest possible score being five. Computing the mode for the ratings given by all participants, see Table 2, we observed that, similar to the relevance of the topics, the majority rated the usefulness of subtopics as four in the case of Bing Chat. However, in the case of ChatGPT 4.0, the usefulness of subtopics was rated by the majority of participants as four, in contrast to the rating of five for the relevance of topics.

Table 4 summarizes the average participant rating per domain regarding the usefulness of the generated subtopics from the PTM questions.

**Table 4:** Average ratings per domain provided by each participant regarding the usefulness of subtopics for both Bing Chat and ChatGPT 4.0.$P_{x}$ stands for participant x.

Domains	$P_{1}$	$P_{2}$	$P_{3}$	$P_{4}$	$P_{5}$	$P_{6}$	$P_{7}$	Total (AVG)
**Bing Chat**								
AVG Diagnosis	4	5	4	4.5	3	4	3.5	4
AVG Emergency Recognition & Measures	4.5	4.5	4	4	3	4.5	4	4.1
AVG Therapy	5	4	4	5	3	3.5	5	4.2
AVG Health Promotion, Prevention	3.5	5	4	4.5	4	5	4	4.3
AVG Structure and Function	4	4.5	4	3.5	3.5	–	4.5	4
AVG Ethics, History, Law	4.5	5	4	5	3	–	3	4.1
AVG Medical-Scientific Skills	4.5	5	3.5	4	2.5	4.5	3	3.9
AVG Diagnostic	4.5	4	4.5	3.5	3	–	4.5	4
AVG Pathogenesis, Pathomechanisms	4	5	4.5	3	3.5	–	4.5	4.1
**ChatGPT 4.0**								
AVG Diagnosis	4.5	5	3.5	4	4	4	4.5	4.2
AVG Emergency Recognition & Measures	4.5	5	2.5	3	3.5	4.5	4	3.9
AVG Therapy	4.5	5	4	5	3.5	4.5	4.5	4.4
AVG Health Promotion, Prevention	4.5	5	3.5	5	4	5	4.5	4.5
AVG Structure and Function	4.5	5	4	4.5	3.5	–	4	4.3
AVG Ethics, History, Law	4.5	5	3.5	4	3.5	–	5	4.3
AVG Medical-Scientific Skills	4	5	4	4.5	3.5	4.5	4.5	4.3
AVG Diagnostic	4	5	4.5	4	3.5	–	4.5	4.3
AVG Pathogenesis, Pathomechanisms	4.5	5	4.5	4	3.5	–	4.5	4.3

Overall, the subtopics in seven out of nine domains, “Diagnosis”, “Therapy”, “Health Promotion, Prevention”, “Diagnostic”, “Structure and Function”, “Ethics, History, Law” and “Pathogenesis, Pathomechanisms”, received an overall average rating of four or above from all participants. Similar to the ratings for the relevance of topics, in the domain “Emergency Recognition and Measures”, Bing Chat received an average rating of four or above from all participants, while ChatGPT 4.0 received a rating below four. The difference in scores between the two methods was 0.2. In the domain “Medical-Scientific Skills” , the ratings were similar to those for the relevance of topics. On average, Bing Chat received a score of 3.9, while ChatGPT 4.0 received a score of 4.3. With a difference of 0.4, the subtopics generated by ChatGPT 4.0 were more preferred. Overall, based on the ratings, ChatGPT 4.0 generated less satisfactory results for both topics and subtopics in the domain “Emergency Recognition and Measures”, while Bing Chat did so for the domain “Medical-Scientific Skills”.

*Assessing participants’ LLM preference.* Asked to decide which method performed better, see Table 5, considering both the topics and subtopics, five participants, including cases where participants found both methods equally good, preferred ChatGPT 4.0 over Bing Chat, while only two participants preferred Bing Chat.

**Table 5:** Participant preference between Bing Chat and ChatGPT 4.0 $P_{x}$ stands for participant x.

	Bing Chat	ChatGPT 4.0	Both	Neither
$P_{1}$	7	6	5	0
$P_{2}$	2	12	4	0
$P_{3}$	6	2	10	0
$P_{4}$	6	9	3	0
$P_{5}$	4	10	2	2
$P_{6}$	0	1	6	0
$P_{7}$	7	9	1	1

*Assessing usefulness of LLMs for content-based feedback generation.* Five of the participants were open to using LLMs for generating content-based feedback. Three out of these five participants were willing to use them regularly, while the other two participants were open to using LLMs occasionally. One participant, however, is not inclined to use LLMs at all.

## Discussion

At the beginning of our manuscript, we posed two research questions that we aimed to address regarding the generation of content-based feedback using LLMs.

With *RQ 1* we aimed to compare the generation of content-based feedback by two prominent LLMs. This comparison involved performing data analysis on the generated feedback and administering a survey to medical practitioners and educators familiar with PTM exams. We prescribed a very specific structure for the output of our prompts. Both models were prompted multiple times in one session by using the structurally same prompt, while solely replacing the question for which content-based feedback was asked for. We applied this process to examine whether the LLMs can generate consistently structured output following our prescriptions. We found that the format was strictly enforced by both LLMs without any variations across the generated outputs. Giving us a first indication on the reliability of the generated outputs. In a precursor study and only on a few occasions, ChatGPT did not generate the expected output, when prompted excessively within a single session. Therefore, we limited sessions to save maximum of twelve consecutive prompts within a single session. The data analysis revealed both similarities and differences in the generated outputs by the two LLMs. By computing the cosine similarity between the topics, subtopics, and the entire output (including both topics and subtopics) for each domain we aimed to provide an inter-model comparison, regarding the repeatability between the wording in the generated outputs of the two models. Our results showed some degree of similarity in the generated responses. However, the similarity was not substantial enough to conclude that the outputs are identical. In certain domains (see Table 1), the topics were more similar than the subtopics, and vice versa. The average similarity across domains for both topics and subtopics ranged between 0.57 and 0.66, slightly higher than the average scores for topics and subtopics considered separately. This we explain by the overlap of vocabulary between the topics and subtopics, thus yielding higher similarity scores. These scores suggest that there is a consistency in the generated responses. However, the models also exhibit variability in their responses and in the order of the generated topics, suggesting that the models weight slightly differ in how they weight the importance of the topics. To further investigate the appropriateness of the generated content for use as feedback, we had medical practitioners and educators closely associated with the PTM evaluate a subset of the content-based feedback. The majority of survey participants were medical practitioners. Compared to medical students, practitioners can more accurately evaluate the correctness and usefulness of the output due to their medical experience. This step was necessary to assess whether the LLMs generate incorrect content, commonly known as hallucinations. The survey results showed no definitive preference for any of the LLMs, and none of the participants remarked that the generated outputs were hallucinated. Both LLMs were highly rated across most domains. In a few domains, the LLMs showed slightly less satisfying results, nevertheless the topics and subtopics in those cases were not rated poorly. We conclude that both LLMs can effectively generate content-based feedback, each with its own strengths and weaknesses. ChatGPT 4.0 generally produces slightly better feedback, while Bing Chat offers the advantage of being free to use at the time of this manuscript’s writing.

*RQ2* aims to assess whether LLMs can effectively extract important content-based feedback from PTM questions. To evaluate this, we utilized participants’ answers from the same survey used in RQ1. Participants needed to evaluate the relevance of topics and the usefulness of subtopics on a Likert scale. Furthermore, they were asked to assess whether they preferred the output from one of the models, both, or neither. Additionally, participants were given the option to express concerns and observations regarding the use of LLMs in the context described in the manuscript. In some cases, such as “Emergency Recognition and Measures” for ChatGPT 4.0 and “Medical-Scientific Skills” for Bing Chat, one model was slightly more preferred compared to the other. Examining the generated output for these cases, we observed that the covered content was similar, however the preferred model captured better the intended focus of the question. For example, a question from the domain “Emergency Recognition and Measures” was asking about the measure that needs to be taken given a specific diagnosis. The preferred model included the expected measure as a topic, while the lower rated model included the measure as a subtopic. This implies the need for more precise refinement of the prompts within these domains, despite the overall ratings indicating that the generated topics and subtopics are not considered useless. Overall, all six participants who completed the survey agree on the necessity of reviewing the generated output. This suggests that they would like to refine or correct the generated feedback. Nevertheless, refining and correcting parts of the feedback is less time-consuming and challenging than generating new feedback from scratch. Among the participants, five are open to using LLMs for generating content-based feedback. Three out of these five participants are willing to use LLMs regularly, while the other two are open to using them occasionally. However, one participant expressed no inclination to use LLMs at all. This participant justified their decision by explaining that while the output generated by LLMs is impressive, it does not always fully capture the intended focus of the questions, which may not necessarily align with the topics covered. We argue however, that capturing the intended focus of the question is challenging even for humans. Exam questions often cover multiple topics, and determining the intended focus can be subjective, varying depending on each individual’s interpretation of the question. However, it’s important to consider that students who answered a specific exam question incorrectly may lack knowledge or have deficits in the topics covered by that question. Nonetheless, the generated content-based feedback can still be valuable, providing them with a starting point for what to revise or learn next. Since the other participants reported high values regarding the topic and subtopics of all questions, we interpret the fact that they did not leave any qualitative feedback as them being generally satisfied with the generated content. We conclude, based on the performed survey, that medical practitioners and educators are open to utilizing LLMs in the form of an assistance and find them useful for generating content-based feedback from PTM questions.

Compared to literature utilizing LLMs in education for generating medical multiple-choice questions^17^, providing feedback on written assignments^19^, and assisting students in learning programming^18^, we also see LLMs as a valuable asset in generating content-based feedback from multiple-choice questions. In a summative assessment, educators can provide students with a clearer summary of their skills and knowledge by linking the scores they achieved in exams to the topics and subtopics in the feedback. Such feedback can help students more easily understand their exam scores, as content-based feedback gives detailed insight into areas where a student lacks knowledge, rather than simply marking a question as wrong. In our case, this content-based feedback is planned to be integrated into the PTM in the future. Up until now, students received more general information about their answers, such as whether a question was correctly answered, and from which domain, subject, or organ system the question originated. By providing more detailed information about the topics related to each question, it becomes easier for students to address knowledge gaps, especially since different topics are covered in different semesters. This allows students to gain an understanding of the exam content without revealing the full questions-important especially in cases where questions are periodically reused. Students can use the topics and subtopics related to PTM questions as a tool for revision. Students can use the subtopics as a starting point to identify what is important to study. For example, a topic might be an illness, and a subtopic could be its symptoms, with a few examples. Students can extend the list of symptoms or add further subtopics as they deepen their knowledge over time. Furthermore, students can better understand how different topics relate to each other since a single question may address multiple topics. For example, a diagnosis topic might also involve related topics, such as therapy or medication for the diagnosis. For educators the content-based feedback can be useful since it can assist them in generating feedback faster, since they do not have to formulate it from scratch. Once generated and proofread and in some cases refined, the feedback can be stored in a database and reused in later PTMs.

Though not perfect, we see the potential of LLMs in generating content-based feedback on exam questions, especially in cases where educators do not have the necessary time or training to generate qualitative feedback, or when the number of test questions or students prevents the generation of feedback in bulk.

## Limitations

There are several potential limitations in our study. Firstly, our primary evaluation method relied on the assessments from the survey we conducted. LLMs have been demonstrated to perform well across various application domains^28^. Thereby, LLMs can be used to answer both multiple-choice as well as open-ended questions. So far, LLMs are among others known to produce precise answers to restricted multiple-choice questions. In the context of the PTM, this has been evaluated by Friederichs et al.^30^ where they found that, in the meantime outdated, ChatGPT LLM could answer two-thirds (66%) out of 400 PTM questions correctly. Here, however we use an LLM to consume a combination of multiple-choice question and its correct answer in order to generate an open-ended feedback in the form of topics and subtopics detailing the question and its answer. Evaluating such open-ended outputs is typically harder than those generated for a structured multiple-choice question and we opted for a survey involving experts assessing the performance of the generated output. Accordingly, we consider our LLM task to be harder than answering a restricted multiple-choice question but conclude based on the assessment of our survey experts that the generated topics overall matched their expectations. Nonetheless, the generated feedback was not given the best possible rating across all participants and more training data, evolved architectures and possibly a context adaptation of the LLM may further increase the acceptance by the PTM survey participants. Given the specific field for which we aimed to generate feedback, we argue that our survey approach provides the most reliable means to assess the correctness and usefulness of the generated feedback. Secondly, we acknowledge the limitation of having a small sample size of only six participants in our survey, which may compromise the representativeness of the findings. This we explain with the subject matter for which we want to generate content-based feedback. Therefore, we needed participants with the requisite expertise and knowledge to evaluate the generated feedback from the two LLMs under examination, thus limiting our participant pool. However, despite the small sample size, we observed fairly consistent opinions regarding the use of LLMs for the task explored in this manuscript. Thirdly, our survey was constructed using a subset of eighteen questions from a larger pool of 200 PTM exam questions. While the size of this set could clearly be larger, it was curated in a way to represent equal amount of randomly selected PTM questions from the nine domains present in the PTM question database. This approach allowed us to assess the capabilities of the LLMs across various domains and uncover potential limitations in their performance. Due to their probabilistic nature, LLMs are typically associated with limitations, such as generating hallucinated content. While this is an important consideration when working with LLMs, our primary focus in this manuscript was to evaluate whether these models are capable of generating plausible feedback from a limited source of textual information, such as PTM questions. To address the possibility of hallucination, we administrated a survey to medical practitioners and educators. Participants could express their concerns and observations regarding the generated content-based feedback. One participant noted that while the models performed well diagnostically, they sometimes struggled to grasp the specific intent of a question, particularly when the question focused on immediate action (emergency measures) rather than general diagnosis. Since the other participants reported high values regarding the topics and subtopics of all questions, we interpret the fact that they did not leave any qualitative feedback as them being generally satisfied with the generated content. We did not perform intra-model comparison assessing the consistency and repeatability of output generated by one model given the exact same prompt, which can be viewed as a further limitation. In an upfront exploration, we found both models to be very repeatable in the content of their generated outputs while para-phrasing the actual answer. Based on this observation and the fact that it is very hard to judge the equivalence of two generated outputs beyond a character-wise similarity, we decided against an intra-model comparison involving multiple requests with the exact same prompt. Instead, we opted for an inter-model comparison in between two seminal LLM instances based on the same fundamental GPT architecture. More specifically, we computed cosine similarity scores between the generated outputs of both models for each question. We observed that while the outputs were different in some extend they were also similar, suggesting that they cover the same focus in both cases. Lastly, a possible limitation is the specific versions of the LLMs examined in this manuscript. Given the rapid development and improvement of LLMs in recent years, we anticipate that future versions will likely exhibit enhanced performance. Nevertheless, the goal of this manuscript is to explore the feasibility of using current LLMs for generating feedback from PTM multiple-choice questions.

## Conclusions

Our findings demonstrate that LLMs can generate content-based feedback from PTM questions, marking the first automated generation of feedback for multiple-choice PTM questions. We conclude that both examined LLMs, i.e., ChatGPT 4.0 and Bing Chat, performed similarly, with neither showing significant deficits in generating feedback from PTM questions. On the contrary, both received an average score of above 4 in regard to the relevance of topics and usefulness of subtopics. However, each LLM has its own advantages and disadvantages: one produces slightly better feedback, as indicated by our survey results, while the other is free to use. Though LLMs are known to generate hallucinated content, we did not find any evidence of this phenomena in our study. Nevertheless, we consider content-based feedback generated by the two examined LLMs as a valuable addition to traditional right-or-wrong feedback, aiding educators in exam preparation and grading. When used correctly, their integration can positively influence and be used as an aid to guide the study path of not only medical students, but also students in general. A potential integration of such LLMs can be especially useful for educational institutions with a shortage of teachers or lecturers, and in cases, where lessons primarily take place online, without direct or limited contact to educators. In a future work of ours, we plan to integrate this type of feedback in a set of multiple runs of the PTM and evaluate its effectiveness on students’ performance and motivation.

## Supplementary Information

Supplementary Information.

## Data Availability

The datasets generated during and/or analyzed during the current study are not publicly available for data security reasons but are available from the corresponding author on reasonable request and after approval of the Progress Test cooperation partners and an extended ethical approval.
