# Supplementary material for: Leveraging large language models to construct feedback from medical multiple-choice Questions
Source: Sci Rep. 2024 Nov 13;14:27910. doi: 10.1038/s41598-024-79245-x (PMC11561272; doi:10.1038/s41598-024-79245-x)

Supplementary material

Article Title: Leveraging Large Language Models to Construct Feedback from Medical Multiple-Choice Questions

Journal Name: Scientific Reports

1 Guidelines of the prompting process

At the time of writing this paper, an OpenAI account for ChatGPT was required. Bing Chat was utilized on a Windows computer using the Edge browser.

1. Use the prompt shown in Fig. 2 from the paper.
2. Prepare an example question and describe how you envision the output to look; you may reference the example provided in Fig. 2.
3. Provide a test question for which you would like to generate content-based feedback, similar to the example you provided.

Mihaela Tomova
Technische Universität Ilmenau, 98693 Ilmenau, Germany
E-mail: mihaela-todorova.tomova@tu-ilmenau.de

Iván Roselló Atanet
Charité - Universitätsmedizin Berlin, corporate member of Freie Universität Berlin and Humboldt Universität zu Berlin, AG Progress Test Medizin, Charité- platz 1, 10117 Berlin, Germany.

Victoria Sehy
Charité - Universitätsmedizin Berlin, corporate member of Freie Universität Berlin and Humboldt Universität zu Berlin, AG Progress Test Medizin, Charité- platz 1, 10117 Berlin, Germany.

Miriam Sieg
Charité - Universitätsmedizin Berlin, corporate member of Freie Universität Berlin and Humboldt Universität zu Berlin, AG Progress Test Medizin, Charité- platz 1, 10117 Berlin, Germany.

Maren März
Charité - Universitätsmedizin Berlin, corporate member of Freie Universität Berlin and Humboldt Universität zu Berlin, AG Progress Test Medizin, Charité- platz 1, 10117 Berlin, Germany.

Patrick Mäder
Technische Universität Ilmenau, 98693 Ilmenau, Germany
Faculty of Biological Sciences, Friedrich Schiller University, 07745 Jena, Germany

2 Survey questions assessing the generated LLM responses and the LLM's usefulness and usability for generating content-based feedback from medical multiple-choice questions

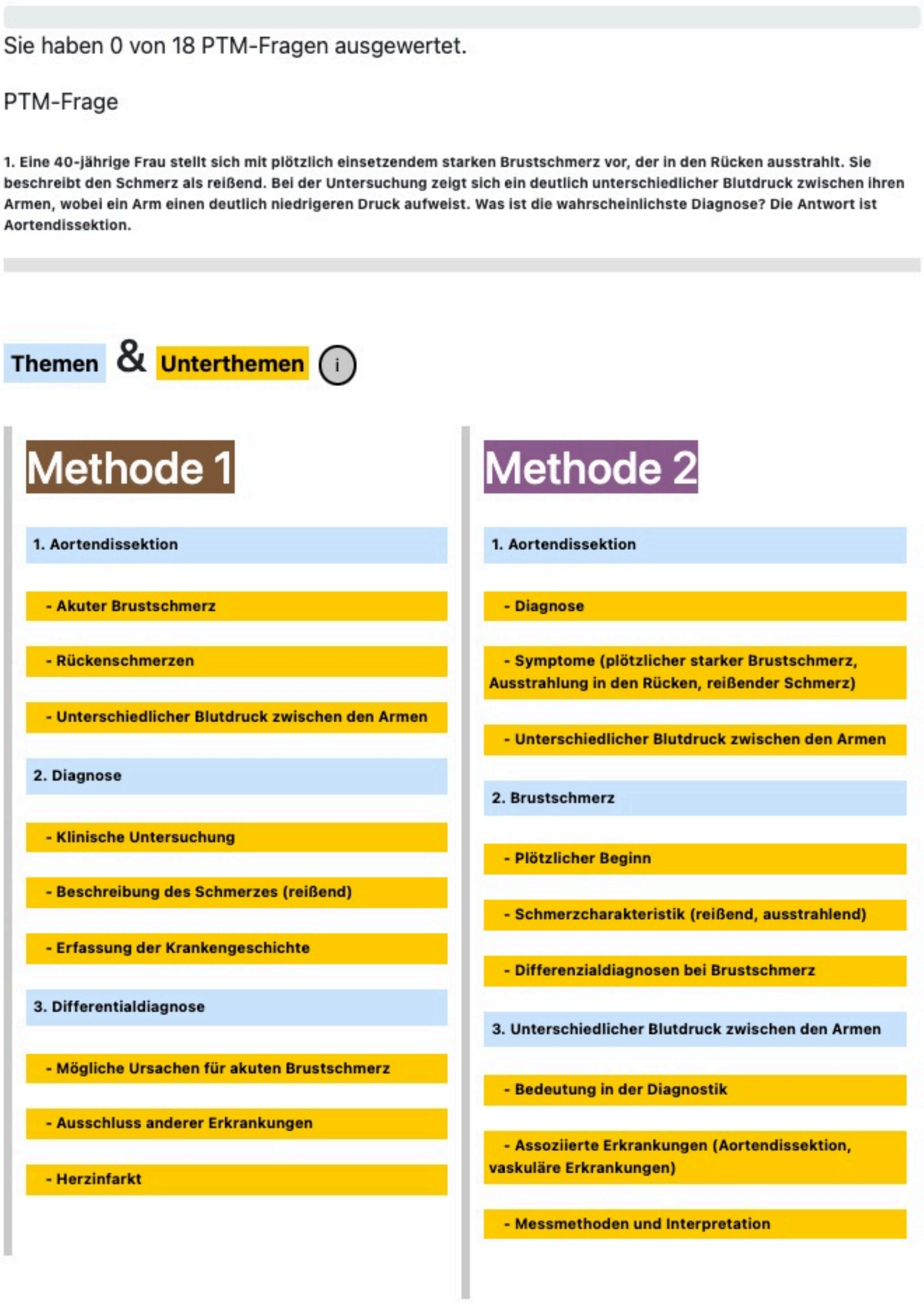

Fig. 1 An example of a PTM question [2] and generated content-based feedback by Bing Chat (Method 1) and ChatGPT 4.0 (Method 2).

[2] The example question is generated by AI due to copyright reasons of the original PTM questions

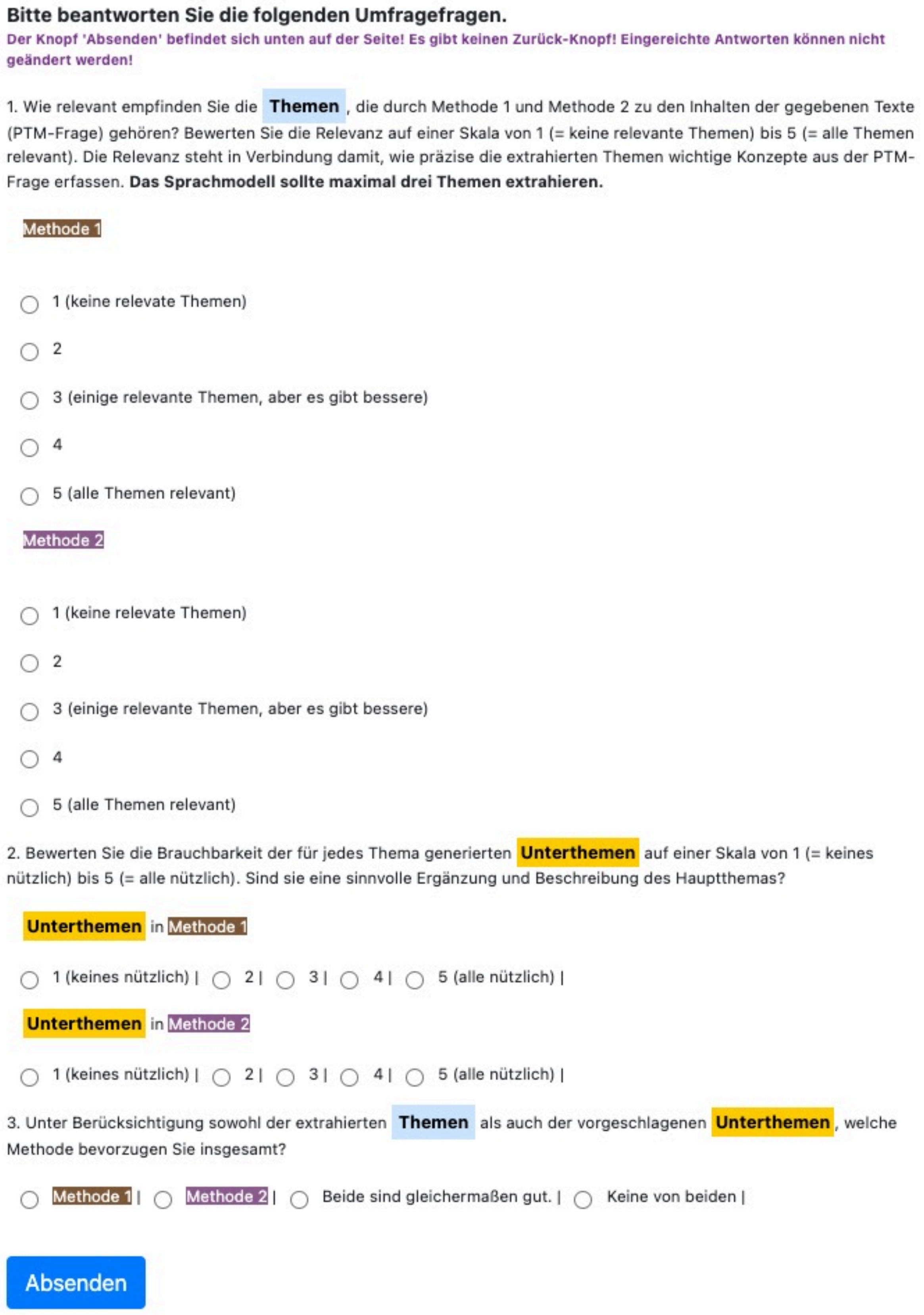

Bitte beantworten Sie die folgenden Umfragefragen.

Der Knopf 'Absenden' befindet sich unten auf der Seite! Es gibt keinen Zurück-Knopf! Eingereichte Antworten können nicht geändert werden!

1. Wie relevant empfinden Sie die **Themen** , die durch Methode 1 und Methode 2 zu den Inhalten der gegebenen Texte (PTM-Frage) gehören? Bewerten Sie die Relevanz auf einer Skala von 1 (= keine relevante Themen) bis 5 (= alle Themen relevant). Die Relevanz steht in Verbindung damit, wie präzise die extrahierten Themen wichtige Konzepte aus der PTM-Frage erfassen. **Das Sprachmodell sollte maximal drei Themen extrahieren.**

Methode 1

- 1 (keine relevate Themen)
- 2
- 3 (einige relevante Themen, aber es gibt bessere)
- 4
- 5 (alle Themen relevant)

Methode 2

- 1 (keine relevate Themen)
- 2
- 3 (einige relevante Themen, aber es gibt bessere)
- 4
- 5 (alle Themen relevant)

2. Bewerten Sie die Brauchbarkeit der für jedes Thema generierten **Unterthemen** auf einer Skala von 1 (= keines nützlich) bis 5 (= alle nützlich). Sind sie eine sinnvolle Ergänzung und Beschreibung des Hauptthemas?

Unterthemen in Methode 1

1 (keines nützlich) | 2 | 3 | 4 | 5 (alle nützlich) |

Unterthemen in Methode 2

1 (keines nützlich) | 2 | 3 | 4 | 5 (alle nützlich) |

3. Unter Berücksichtigung sowohl der extrahierten **Themen** als auch der vorgeschlagenen **Unterthemen**, welche Methode bevorzugen Sie insgesamt?

Methode 1 | Methode 2 | Beide sind gleichermaßen gut. | Keine von beiden |

Absenden

Fig. 2 Survey questions about the assessment of the generated LLM responses.

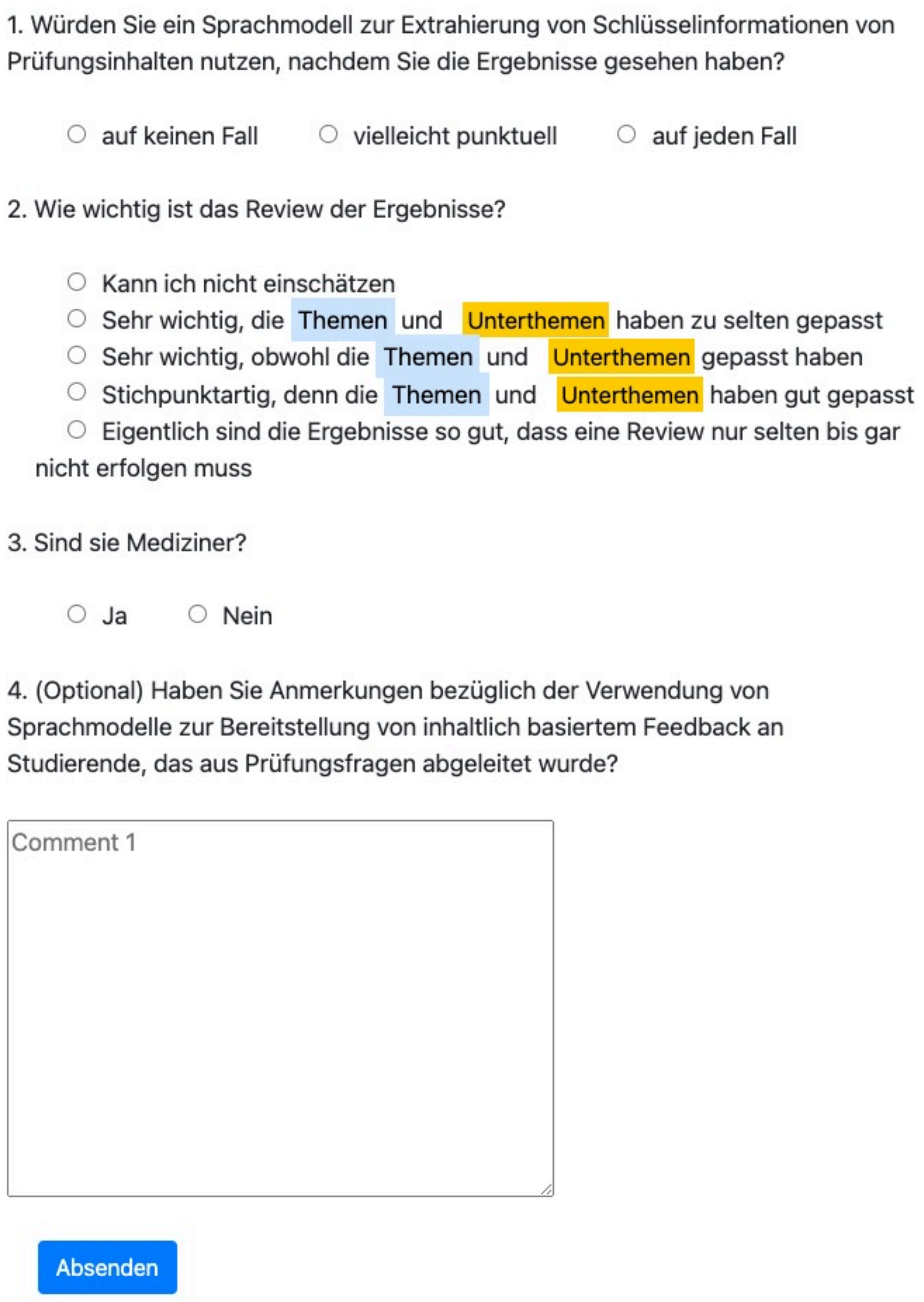

1. Würden Sie ein Sprachmodell zur Extrahierung von Schlüsselinformationen von Prüfungsinhalten nutzen, nachdem Sie die Ergebnisse gesehen haben?

- ○ auf keinen Fall ○ vielleicht punktuell ○ auf jeden Fall

2. Wie wichtig ist das Review der Ergebnisse?

- ○ Kann ich nicht einschätzen
- ○ Sehr wichtig, die Themen und Unterthemen haben zu selten gepasst
- ○ Sehr wichtig, obwohl die Themen und Unterthemen gepasst haben
- ○ Stichpunktartig, denn die Themen und Unterthemen haben gut gepasst
- ○ Eigentlich sind die Ergebnisse so gut, dass eine Review nur selten bis gar nicht erfolgen muss

3. Sind sie Mediziner?

- ○ Ja ○ Nein

4. (Optional) Haben Sie Anmerkungen bezüglich der Verwendung von Sprachmodelle zur Bereitstellung von inhaltlich basiertem Feedback an Studierende, das aus Prüfungsfragen abgeleitet wurde?

Comment 1

Absenden

Fig. 3 Survey questions about the background of the participants and their general perception of the LLMs.

3 Wordclouds generated from subtopics per domain (in German)

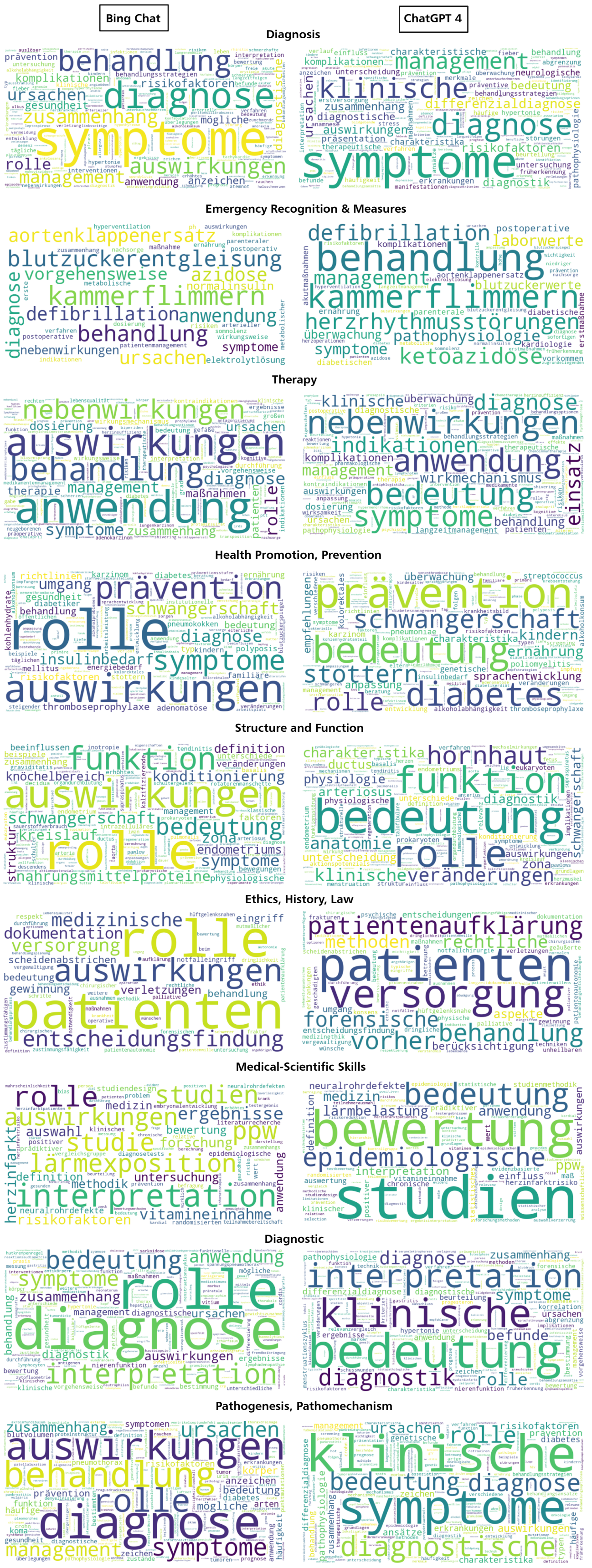

Supplement: Supplementary file 1 — Supplementary Information. [file 41598_2024_79245_MOESM1_ESM.pdf]
